# Developmental constraint through negative pleiotropy in the zygomatic arch

**DOI:** 10.1186/s13227-018-0092-3

**Published:** 2018-01-27

**Authors:** Christopher J. Percival, Rebecca Green, Charles C. Roseman, Daniel M. Gatti, Judith L. Morgan, Stephen A. Murray, Leah Rae Donahue, Jessica M. Mayeux, K. Michael Pollard, Kunjie Hua, Daniel Pomp, Ralph Marcucio, Benedikt Hallgrímsson

**Affiliations:** 1 Department of Anthropology, Stony Brook University, Stony Brook, NY USA; 2 Alberta Children’s Hospital Institute for Child and Maternal Health, University of Calgary, Calgary, AB Canada; 3 The McCaig Bone and Joint Institute, University of Calgary, Calgary, AB Canada; 4 Department of Cell Biology and Anatomy, University of Calgary, Calgary, AB Canada; 5 Program in Ecology Evolution and Conservation Biology, University of Illinois, Urbana, IL USA; 6 The Jackson Laboratory, Bar Harbor, ME USA; 7 Department of Molecular Medicine, The Scripps Research Institute, La Jolla, CA USA; 8 Department of Genetics, University of North Carolina Medical School, Chapel Hill, NC USA; 9 The Orthopaedic Trauma Institute, Department of Orthopaedic Surgery, UCSF School of Medicine, San Francisco, CA USA

**Keywords:** Craniofacial, Skull, Micro-computed tomography, Morphometrics, Integration, QTL analysis, Diversity Outbred, RT-PCR, *Mus musculus*

## Abstract

**Background:**

Previous analysis suggested that the relative contribution of individual bones to regional skull lengths differ between inbred mouse strains. If the negative correlation of adjacent bone lengths is associated with genetic variation in a heterogeneous population, it would be an example of negative pleiotropy, which occurs when a genetic factor leads to opposite effects in two phenotypes. Confirming negative pleiotropy and determining its basis may reveal important information about the maintenance of overall skull integration and developmental constraint on skull morphology.

**Results:**

We identified negative correlations between the lengths of the frontal and parietal bones in the midline cranial vault as well as the zygomatic bone and zygomatic process of the maxilla, which contribute to the zygomatic arch. Through gene association mapping of a large heterogeneous population of Diversity Outbred (DO) mice, we identified a quantitative trait locus on chromosome 17 driving the antagonistic contribution of these two zygomatic arch bones to total zygomatic arch length. Candidate genes in this region were identified and real-time PCR of the maxillary processes of DO founder strain embryos indicated differences in the RNA expression levels for two of the candidate genes, *Camkmt* and *Six2*.

**Conclusions:**

A genomic region underlying negative pleiotropy of two zygomatic arch bones was identified, which provides a mechanism for antagonism in component bone lengths while constraining overall zygomatic arch length. This type of mechanism may have led to variation in the contribution of individual bones to the zygomatic arch noted across mammals. Given that similar genetic and developmental mechanisms may underlie negative correlations in other parts of the skull, these results provide an important step toward understanding the developmental basis of evolutionary variation and constraint in skull morphology.

**Electronic supplementary material:**

The online version of this article (10.1186/s13227-018-0092-3) contains supplementary material, which is available to authorized users.

## Background

The skull is a complex structure that supports and protects tissues critical for survival, including the brain, sense organs, and masticatory apparatus. While a wide range of skull morphology has evolved across mammalian species, fundamental patterns of skull bone integration are generally conserved [1–4]. This shared morphological pattern reflects conserved tissue origins [5], ossification patterns [6–8], and strong selective pressure for an adequately integrated and functional head [2, 9]. The same factors that reinforce the development of an integrated head can serve as developmental constraints on the directions that evolution can take [10, 11]. Understanding the genetic basis for this integration and developmental constraint is critical for illuminating how genes and development can influence processes of evolution. Here, we investigate the genetic basis for negative correlations between adjacent skull bones in a large genetically heterogeneous population of mice. These negative correlations may serve as developmental constraints that support overall integration of the head while also allowing for significant variation in the relative size of individual bones within subregions. Identifying genetic factors underlying this type of developmental constraint is important for understanding the basis of morphological integration and will illuminate critical connections between developmental and evolutionary processes.

Random pairs of linear distances across the skull are expected to display positive correlations driven by the overall growth of the integrated head. Negative correlations between raw linear distance measurements are rare and can be evidence of scale-independent negative pleiotropy, which exists when genetic variation leads to an opposite phenotypic effect on two traits [10, 12]. Within the craniofacial skeleton, a pair of negatively correlated and adjacent bone lengths provides a relatively simple system within which to search for genes underlying negative pleiotropy. Although not as commonly discussed, negative pleiotropy is fundamentally different than antagonistic pleiotropy, which occurs when a genetic factor contributes a positive fitness effect for at least one trait and a negative fitness effect for at least one other trait [13–15]. The concept of negative pleiotropy does not require either trait to be associated with a fitness effect. However, the existence of negative pleiotropy itself might have either positive or negative fitness effects, depending on the evolutionary context.

A previous analysis of adult skull variation indicated that linear dimensions of the cranial vault, zygomatic arch, and cranial base vary strongly among the eight inbred mouse founder strains of the Collaborative Cross (CC). Furthermore, that analysis indicated that pairs of bones within each of these regions display negative correlations in relative size [16]. Here, we explicitly tested whether pairs of adjacent linear distances within cranial vault, zygomatic arch, and cranial base are negatively correlated. In cases where they were, we performed genome-wide mapping in a large heterogeneous population of Diversity Outbred (DO) mice to identify genomic regions driving negative pleiotropy. The DO mice are derived from the CC founder strains. Each mouse carries a high degree of heterozygosity and a unique combination of alleles [17, 18], which is ideal for high-resolution genetic mapping. Although genome-wide mapping has been performed on DO mice for other phenotypes such as blood measurements and body composition [17–19], this is the first study that addresses craniofacial morphology.

Previous quantitative trait locus (QTL) analyses of mouse craniofacial variation have illustrated the ubiquity of pleiotropy in the genetics of craniofacial form [20–22] and identified candidate genes associated with variation in multivariate measures of craniofacial shape in a variety of mouse populations [23–27], including another outbred mouse sample [28]. The DO mice may prove more valuable than previously analyzed mouse crosses or backcrosses, because they represent a strongly genetically heterogeneous population that has been outbred for more than 10 generations, leading to relatively high genomic mapping resolution. In addition, haplotype variation at a given SNP can be tied back to the complete genomic sequence of eight diverse inbred founder strains.

Within our mapping results, we expect that a locus driving negative pleiotropy will have a significant haplotype effect on the linear distances of each adjacent bone. Furthermore, we expect that variation in founder strain haplotypes at this locus will be associated with opposite phenotypic effects for each bone (i.e., one bone gets longer when the other gets shorter). Third, if this locus represents a developmental constraint on the morphology of the combined length of both bones, we expect that local haplotype variation will have no effect on overall regional length. Identifying a genomic region associated with negative pleiotropy and candidate genes within it is a critical first step in understanding an important genetic and developmental basis for both developmental constraint and variation in the relative contribution of bones to regional skull morphology. Although our analyses are regionally specific, identifying the general mechanisms underlying negative pleiotropy of adjacent skeletal elements is an important step toward understanding the developmental basis for evolutionary change in the skull.

## Methods

### Adult and embryonic sample

Our measurements of the Collaborative Cross (CC) founder strains and their F1 crosses derive from craniofacial landmarks collected from 1211 specimens for a previous analysis [16]. The Diversity Outbred (DO) mice are the result of multiple generations of random outcrossing of 175 breeding pairs from partially inbred CC lines [18] at the Jackson Laboratory (JAX; Bar Harbor, ME). Our primary sample consisted of mice raised at the University of North Carolina (UNC), and at JAX. The 287 adult specimens raised at UNC were male and female sibling pairs from outbreeding generation 10 that were raised in previously described conditions [19, 29] under approval and conduced in accordance with the guidelines set forth by the Institutional Animal Care and Use Committee (IACUC) at the University of North Carolina at Chapel Hill. The 277 adult specimens raised at Jackson Labs (JAX IACUC #99066) were females of outbreeding generations 9, 10, and 15.

A sample of 472 DO mice of generations 19, 21, and 23, raised at the Scripps Research Institute (IACUC #08-0150-3), were used to validate relevant QTLs identified by genome-wide association in our primary sample. This validation sample was chosen because it includes mice from later DO generations with greater recombination, which might allow for the refinement of any validated QTL (i.e., shorter confidence intervals). After collection, adult specimens were stored at −20 °C. Receipt and imaging of specimens from other institutions was conducted in accordance with approved IACUC protocol #AC13-0268 at the University of Calgary.

Embryonic specimens from A/WySnJ (AWS), C57BL/6J (C57), and WSB/EiJ (WSB) inbred backgrounds were collected at the University of Calgary at embryonic day (E) 11.5 and processed as recently described [30]. Briefly, embryos were fixed overnight in PaxGene tissue fix solution (Qiagen, PreAnalytics, cat #765312) then stored at −20 °C in the PaxGene tissue stabilizer solution prepared to manufacturer specification (Qiagen, PreAnalytics, cat #765512). Embryonic collection and processing were conducted in accordance with approved IACUC protocol AC13-0267 at the University of Calgary.

### Linear distances versus relative linear dimensions

**Fig. 1:**
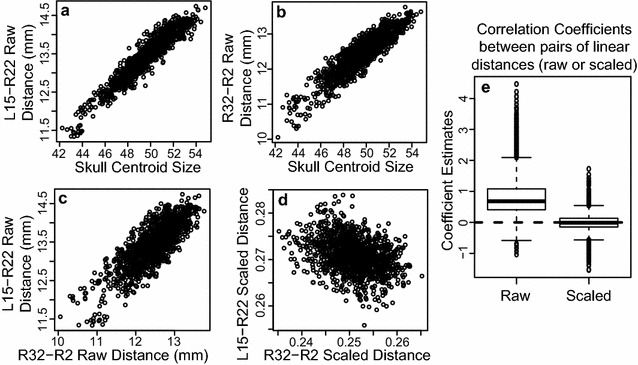
Linear distance correlations and scaling. Plots of two randomly chosen linear distances calculated from our CC founder and F1 sample illustrating the change in correlation direction after scaling measures to overall skull size (centroid size). Plots of raw linear distance **a** L15–R22 and **b** R32–R2 versus skull centroid size. Plots of linear distance L15–R22 versus R32-R2 **c** before and **d** after Procrustes superimposition-based scaling (based on centroid size). **e** Correlation coefficients from 10,000 randomly permuted linear distance pairs calculated from raw linear distances (left) and after (right) Procrustes superimposition and scaling

Negative relationships between the length of bones making up the zygomatic arch, sagittal cranial vault, and posterior cranial base were previously identified among the eight CC founder strains from plots of relative linear dimensions that differ strongly between a given founder strain and several of the other founder strains [16]. Unlike standard linear distances, these relative dimensions were calculated from landmark coordinates after they were scaled to remove overall size variation and then transformed to remove the linear component of static allometry. These steps were necessary in the previous analysis of craniofacial shape between genotypes with a wide range of sizes, including small wild-derived strains and the very large New-Zealand Obese strain. However, because most raw linear distances are positively correlated with head size, removing variation that covaries with overall size can create artefactual negative associations between many of the resulting linear dimensions. For instance, two randomly selected linear distances are both strongly positively correlated with head size (centroid size) (Fig. 1a, b). They are also strongly positively correlated with each other (Fig. 1c). After scaling landmark coordinates underlying these measurements by overall skull size during Procrustes superimposition, variation that is correlated with overall scale is removed, regardless of whether that variation is mechanistically or developmentally related to overall size variation. This frequently leads to an artificial negative correlation between the resulting linear dimensions (Fig. 1d). Correlation coefficients from many linear distance pairs illustrates how scaling during Procrustes superimposition changes an asymmetric distribution of correlation coefficients between raw linear distances into a symmetric distribution of correlation coefficients centered on zero (Fig. 1e). Because this scaling procedure magnifies aspects of negative covariation, we completed our current analysis of potential developmental constraints on adjacent bones using raw linear distances to make certain that our evidence for negative relationships between these skull lengths are genuine.

### Phenotype measurement

Micro-computed tomography (µCT) images of heads were obtained in the 3D Morphometrics Centre at the University of Calgary with a Scanco vivaCT40 scanner (Scanco Medical, Brüttisellen, Switzerland) at 0.035 mm voxel dimensions at 55 kV and 145 µA. Three dimensional coordinates of 54 adult landmarks (8 midline, 46 bilateral), as previously defined [16], were collected by a single observer from minimum threshold defined bone surfaces within Analyze 3D (www.mayo.edu/bir/).

**Fig. 2:**
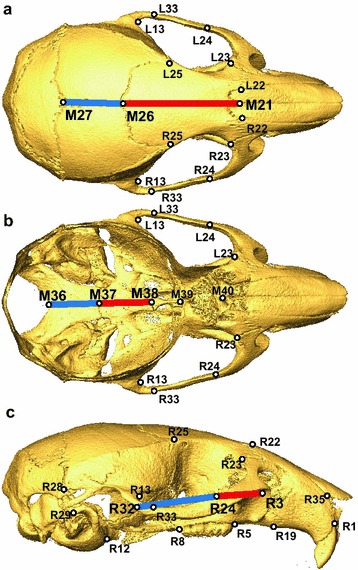
Linear distances for analysis. Pairs of linear distances that were tested for a negative correlation within CC founder/F1 and DO samples, plotted between previously defined craniofacial landmarks [16]. **a** Sagittal cranial vault lengths of the frontal bone (red) and parietal bone (blue) plotted on a superior view, **b** posterior cranial base lengths of the basioccipital (blue) and sphenoid body (red plotted on an interior view, **c** zygomatic arch lengths of the zygomatic bone (blue) and zygomatic process of the maxilla (red) on a lateral view

We calculated linear distances associated with zygomatic arch length, sagittal cranial vault length, and midline cranial base length from raw landmark coordinates collected on both CC founder/F1 specimens and DO specimens (Fig. 2). Full zygomatic arch length was calculated as the linear distance between landmarks L(R)3 and L(R)32, with the length of zygomatic process of the maxilla defined between L(R)3 and L(R)24 and zygomatic bone length defined between L(R)24 and L(R)32. Full cranial vault length was defined between M21 and M27, while the length of the frontal and parietal bones were defined between M21–M26 and M26–M27, respectively. Posterior cranial base length was defined between M36 and M38, while the lengths of the basioccipital and sphenoid bones were defined between M36–M37 and M37–M38, respectively. We completed our analysis of the zygomatic arch lengths on the average of the left and right sides to help control for any stochastic landmarking error or stochastic developmental variation between the left and right sides. We were unable to do this for the other measurements, because they are found along the midline of the skull.

The Pearson’s correlation coefficient (*r*) for each pair of component linear distances (e.g., zygomatic bone length vs. zygomatic process of maxilla length) and between each component linear distance and the overall trait length (e.g., zygomatic bone length vs. total zygomatic arch length) was calculated to identify correlation direction and strength. A *t* test of whether the r is different from 0 was completed for each pair to determine whether their correlation is significant, after Bonferroni correction to account for multiple testing (*α* = 0.0028). The coefficient of determination (*R*^2^) is interpreted as a measure of how much variance in one length in a pair is explained by the other.

### Genotyping and association mapping

Tail biopsies were taken from mice at 6 weeks of age, and DNA was either extracted from the tissue using the QIAGEN DNeasy kit per manufacturer’s instructions or sent to NeoGen GeneSeek for DNA extraction. The primary DO sample was genotyped using the MegaMUGA SNP array [GeenSeek (Neogen), Lincoln, NE] [31], while the validation sample was genotyped using the GigaMUGA array (GeenSeek (Neogen), Lincoln, NE) [32]. We used a subset of 57,977 MegaMUGA SNPs or 120,789 GigaMUGA SNPs that distinguish among the genotypes of the eight CC founder strains and their heterozygous F1 offspring and have a quality tier of 1 or 2 [32]. The probability that each of the eight founder strains contributed to a given SNP maker was calculated for each DO specimen based on array intensity values using the DOQTL package [17] within R [33].

Association mapping was performed using DOQTL [17] for both components and the overall trait length of linear distance pairs that displayed a significant negative relationship within our primary DO mouse sample. First, we completed genome scans for the primary sample using an additive haplotype model for regression of the specimen founder genotype dosage on an individual linear distance, with age at sacrifice and sex as covariates. Peaks indicating regions of the genome where haplotype covaries with a given trait were identified as those with LOD scores above a genome-wide significance threshold determined with permutation tests (1000 iterations, indicating a LOD score ~ 7.76). The support intervals under these significant peaks were identified as continuous regions including 2 LOD scores below the LOD score value of that significant peak. The results of these additive haplotype regressions also indicate which founder haplotypes are associated with positive or negative increases in linear distance length. We completed association mapping using an additive SNP-based regression model [17] across the genomic intervals of interest that were identified in the previous step. A permutation test of the SNP-based regression model across the genome was used to determine the LOD score significance threshold for these tests. Second, to validate significant peaks on chromosome 17, we completed the same association mapping steps with our validation sample for zygomatic arch distances across chromosome 17 (rather than across the whole genome).

### RT-PCR

The E11.5 maxillary process was chosen for RT-PCR because both the maxilla and zygomatic bones are derived from mesenchymal condensations within this process (see “Discussion”), starting at approximately E11.5. Specifically, this age was chosen because it is the approximate time when morphogenesis and differentiation of the facial skeleton begins [34, 35]. Mouse embryos were dissected in ice cold PBS and immediately preserved using the PaxGene tissue system (Qiagen, PreAnalytics cat #765312, 765512). Embryos were fixed overnight in the fix at room temperature with rocking, then transferred to the stabilization buffer and stored at −20 °C. Maxillary processes were subsequently micro-dissected from five A/WySnJ (AWS), five C57BL/6J (C57), and six WSB/EiJ (WSB) embryos that had been stored in PaxGene tissue stabilization buffer and were stored in fresh stabilization buffer at −20 °C until extraction. AWS embryos were used in place of A/J embryos, because they were available at the University of Calgary and because the two are closely related inbred strains.

Following a recently published analysis [30], RNA was extracted using the PaxGene RNA extraction kit (Qiagen, PreAnalytics cat #766134), which includes a DNA removal step. RNA was analyzed using a NanoDrop 1000 (ThermoFisher). While RIN analysis was not performed on these samples, a similar group of samples processed during the same time span (Agilent BioAnalyzer) had RIN scores in the area of 7.9–8.3, which is in line with kit specifications. 500 ng of RNA was converted to cDNA using the Maxima First Strand Kit (ThermoFisher, Cat #K1641) in a 25 µl reaction. Real-time PCR was performed on an Applied Biosystems Quantiflex Studio 6 using standard cycling conditions with the low volume (10 µl) setting. Reactions were performed using the 2× PrimeTime gene expression mastermix from Integrated DNA Technologies with low ROX, PrimeTime assays (*Gapdh*—Mm.PT.39a.1, *Camkmt*—Mm.PT.58.7890215, *Six3os1*—Mm.PT.58.43925739, *Six2*—Mm.PT.58.22007192), an ABI Taqman assay (18s—Mm0477571_s1), and custom *Six3* primers (Probe: 5′-CAAACTTCGCCGATTCTCACCACTGCT-3′, Forward primer: 5′-TCTCTATTCCTCCCACTTCTTGTTG-3′, Reverse primer: 5′-GCCGCTACTCGCCAGAAGTA-3′) [36]. Additional primer sequence details are found in Additional file 1. Normalization was done using the arithmetic average of the deltaCT from *Gapdh* and 18 s RNA runs. Reference genes were selected based on stability from previous experiments and RNAseq data from this region of the face.

Since C57 mice had an intermediate phenotypic effect, mean C57 RNA expression was used as the baseline upon which to compare the expression of all specimens (measured as fold change). One-way ANOVA tests of fold-change values between genotypes were completed for *Camkmt*, *Six2*, *Six3*, and *Six3OS1* using Graphpad Prisim (Version 6) software. If there were differences in expression between genotypes, we looked for similarities between variation in RNA expression and the phenotypic effects of A-strain, C57, and WSB haplotypes on relative zygomatic bone length, which might indicate that variation in expression of these candidate genes is associated with the identified negative correlation in zygomatic bone lengths. This was done by using a post-test for linear trend.

## Results

We explicitly tested whether the lengths of adjacent bones within the cranial base, cranial vault, and zygomatic arch were negatively correlated. While we expected that most linear distances in the skull would be positively correlated, a negative correlation is evidence for a developmental constraint in how component bones (e.g., frontal and parietal bones) contribute to a larger overall trait (e.g., cranial vault length). All component bone lengths were significantly positively correlated with corresponding overall trait lengths (Table 1). The lengths of component bones of the zygomatic arch were negatively correlated within the CC Founder/F1 and DO samples, while cranial vault components were negatively correlated within the DO sample. There was no evidence of a negative association between components of the posterior cranial base.

**Table 1:** Linear distance correlations

Linear distance 1	Linear distance 2	CC founder/F1			DO		
		Pearson’s correlation coefficient (*r*)	*R* ^2^	*r* ≠ 0 *t* test *p* value	Pearson’s correlation coefficient (*r*)	*R* ^2^	*r* ≠ 0 *t* test *p* value
Zygomatic Process of Maxilla (L3–L24)	Zygomatic Bone (L24–L32)	***− 0.232***	***0.054***	***< 0.001***	***− 0.294***	***0.086***	***< 0.001***
Zygomatic Process of Maxilla (L3–L24)	Full Zygomatic Arch (L3–L32)	0.465	0.216	< 0.001	0.620	0.385	< 0.001
Zygomatic Bone (L24–L32)	Full Zygomatic Arch (L3–L32)	0.748	0.560	< 0.001	0.561	0.315	< 0.001
Frontal Bone (M21–M26)	Parietal Bone (M26–M27)	− 0.034	0.001	0.232	***− 0.345***	***0.119***	***< 0.001***
Frontal Bone (M21–M26)	Vault Length (M21–M27)	0.776	0.602	< 0.001	0.622	0.386	< 0.001
Parietal Bone (M26–M27)	Vault Length (M21–M27)	0.603	0.363	< 0.001	0.519	0.270	< 0.001
Basioccipital (M36–M37)	Sphenoid Body (M37–M38)	0.592	0.350	< 0.001	0.333	0.111	< 0.001
Basioccipital (M36–M37)	Posterior Cranial Base (M36–M38)	0.870	0.757	< 0.001	0.786	0.618	< 0.001
Sphenoid Body (M37–M38)	Posterior Cranial Base (M36–M38)	0.912	0.832	< 0.001	0.844	0.713	< 0.001

Bolditalic indicates a significant negative correlation after accounting for multiple testing with Bonferonni correction (*α* = 0.0028)

### Association mapping

**Fig. 3:**
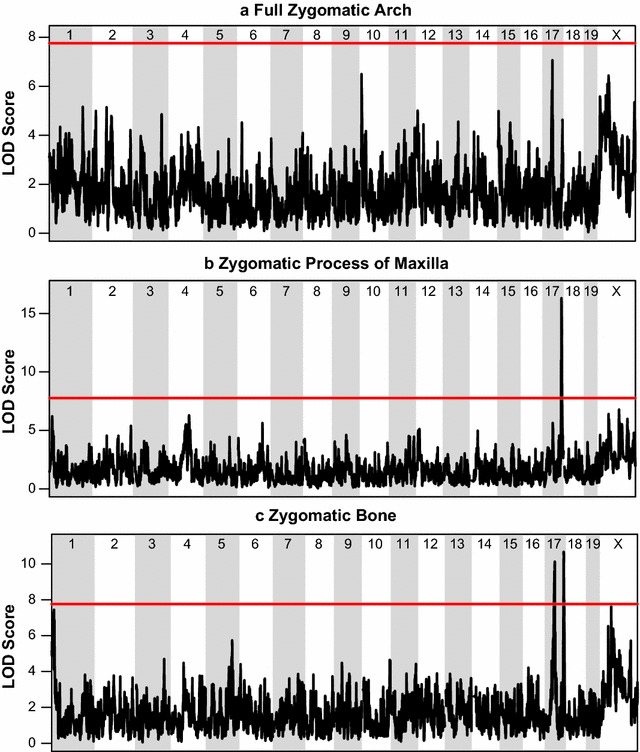
Genome-wide scan results. The results of genome-wide scan using an additive haplotype model to identify genomic regions significantly associated with **a** full zygomatic arch length (landmark 3–33), **b** zygomatic process of the maxilla length (landmark 3–24), and **c** zygomatic bone length (landmark 24–33). The red line is a permutation-based (1000 iterations) significant LOD score of 7.76

**Fig. 4:**
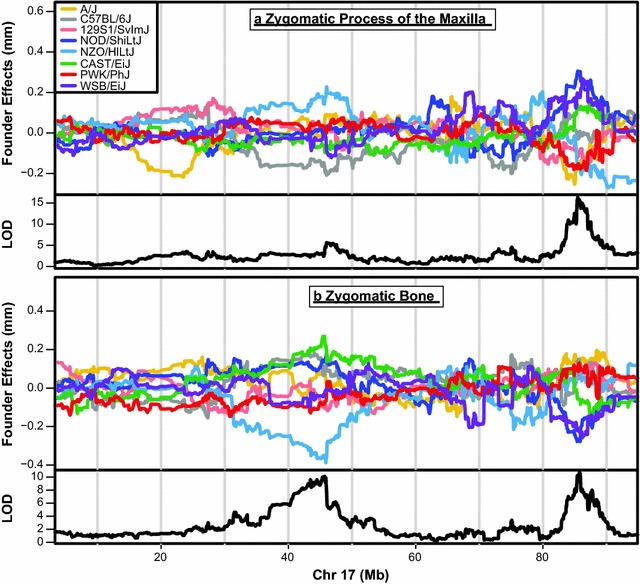
Founder haplotype effects. CC founder strain specific phenotype coefficients (above) and LOD scores from genome-wide scan (below) for significant association between haplotype and **a** zygomatic process of the maxilla length and **b** zygomatic bone length across Chromosome 17. Phenotypic coefficients are the effect of having a certain founder strain genotype at a specific genomic location on a measurement

**Fig. 5:**
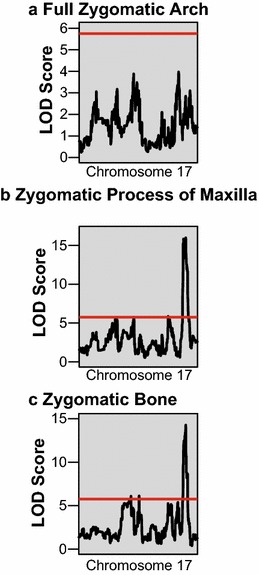
Validation sample chromosome 17 scan results. The results of chromosome 17 specific scan using an additive haplotype model to identify genomic regions significantly associated with **a** full zygomatic arch length (landmark 3–33), **b** zygomatic process of the maxilla length (landmark 3–24), and **c** zygomatic bone length (landmark 24–33). The red line is a permutation-based (1000 iterations) significant LOD score of 5.75

Given the negative correlations between the length of bones contributing to the zygomatic arch and cranial vault, we completed genome-wide association mapping to look for evidence of a genomic region that might drive these correlations based on the mechanism of negative pleiotropy. Our association mapping analysis revealed two intervals on chromosome 17 that were associated with zygomatic bone length variation (42.04829–45.95447; 85.30648–85.88324 Mb), while the second of these intervals (85.30648–85.88324 Mb) was also associated with zygomatic process of the maxilla length variation (Fig. 3b, c). The phenotypic effects of founder haplotypes under the second peak were in opposite directions for the two components (Fig. 4), meaning that a founder haplotype associated with an increase in zygomatic length was also associated with a decrease in zygomatic process of the maxilla length. This closely matches our expectation for a gene underlying negative pleiotropy between two components of a larger trait. Furthermore, this interval displays a significant LOD score for both components but not for total zygomatic arch length (Fig. 3a), which meets our expectation that a gene underlying a developmental constraint on zygomatic arch morphology will have opposite effects on the relative contribution of the components to zygomatic arch length without effecting overall arch length. Chromosome specific association mapping with our validation sample confirmed the second zygomatic peak on chromosome 17 (84.37429–85.86122 for zygomatic bone; 83.71674–85.88897 for zygomatic process of maxilla), the general phenotypic effects of haplotypes under this peak (Additional file 2), and suggested a few other significant peaks on chromosome 17 related to zygomatic arch length (Fig. 5). In addition, a single significant peak on the X chromosome was noted for total cranial vault length (98.220635–100.409455) in our primary sample, but it did not reach significance in our validation sample. This vault length peak was not further pursued in this study because it did not reach significance for either cranial vault component.

**Fig. 6:**
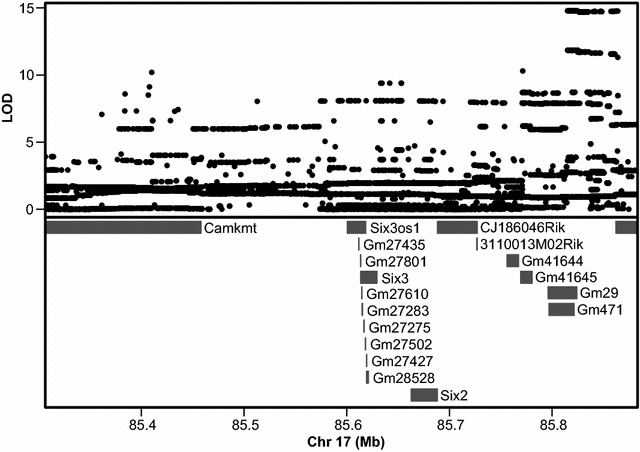
Association mapping results. Results of association mapping under the significant peak that is found in the genome-wide scan results for both zygomatic bone length and zygomatic process of maxilla length

Association mapping across the support interval of the second peak associated with zygomatic arch variation on chromosome 17 (85.30648–85.88324 Mb) indicates there are 19 known or predicted genes in this region (Fig. 6). These include three protein-coding genes (*Six2*, *Six3*, *Camkmt*) and one well-studied non-coding RNA (*Six3os1*). We noted that the WSB and A/J founder haplotypes are associated with opposite phenotypic effects for zygomatic bone and zygomatic process of the maxilla lengths, while the C57 haplotype effect is intermediate (Fig. 4). Therefore, if differences in the expression level of a protein-coding gene were responsible for founder haplotype associated variation in zygomatic bone length, we expected that A-strain and WSB expression levels would be most different, while C57 expression would be intermediate.

### RT-PCR

**Fig. 7:**
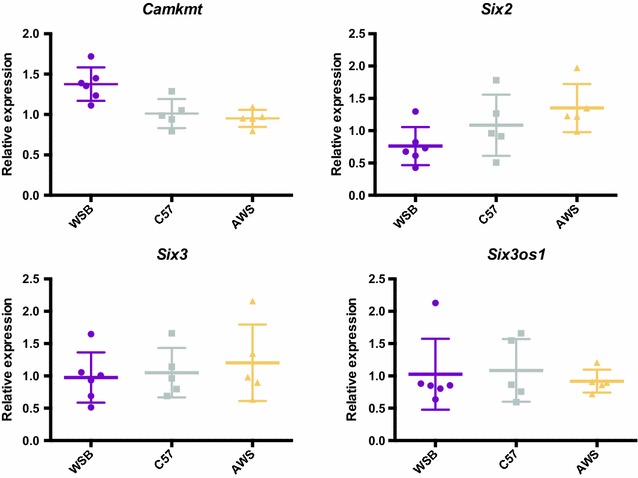
RT-PCR results. RNA expression levels from E11.5 maxillary processes of three inbred mouse genotypes, relative to C57 expression levels

To test whether the expression of these four candidate genes met this expectation, we collected maxillary prominences from embryonic day (E) 11.5 embryos of AWS, C57, and WSB mice, which had been stage matched by tail somite number. We then completed RT-PCR on these tissue samples using three replicates for each sample to quantify RNA expression levels for *Camkmt, Six2*, *Six3*, and *Six3os1*. All fold-change values were compared to the C57 mean as a baseline. One-way ANOVA results indicate genotype identity significantly contributes to *Camkmt* and *Six2* RNA expression (Table 2). In both cases, a post-test for linear trends is significant when genotypes are ordered as WSB, C57, then AWS. WSB displays relatively high mean *Camkmt* RNA expression levels and relatively low *Six2* levels. AWS displays relatively high mean *Six2* levels and intermediate mean *Camkmt* levels (Fig. 7). No significant trends are noted for either *Six3* or *Six3OS1*.

**Table 2:** ANOVA post-test for a linear trend in RNA expression, where the alternate hypothesis is that the level of expression is ordered by genotype with C57 being intermediate to the other two genotypes (AWS and WSB)

Gene	Slope	*R* ^2^	*p* value
*Camkmt*	− 0.2118	0.5009	0.0014**
*Six2*	0.2949	0.3342	0.0237*
*Six3*	0.1141	0.04933	0.426
*Six3OS1*	− 0.05338	0.01178	0.698

Slope of the associated linear model, *R*^2^ indicating how much variation that model explains, and a p-value of the probability that the slope of the linear model equals zero are reported

*Significance at 0.05 level; **Significance at 0.01 level

## Discussion

Within a sample of DO mice, we confirmed negative correlations between the lengths of bones contributing to the cranial vault and found strong evidence for negative pleiotropy between the length of the zygomatic bone and the zygomatic process of the maxilla. A genomic interval on chromosome 17 (85.3–85.9 Mb) met all our expectations for a genetic basis of negative correlation between these adjacent zygomatic arch bones. Specifically, there were significant and opposite haplotype effects on zygomatic and zygomatic process length (Figs. 3a, b, and 4), but no significant haplotype association with overall zygomatic arch length (Fig. 3c). This example of negative pleiotropy shows how skull shape can be conserved while the individual bone contributions to that shape can vary. The development of an integrated skull that fits together well enough for masticatory, sense organ, breathing, and brain function is necessary for survival [1, 11, 12, 37]. Just as with any skull region, the range of possible zygomatic arch phenotypes that allow for proper skull integration and function is limited. Our results illustrated a mechanism of developmental constraint that supports skull integration while allowing for variation in how specific bones contribute to a fundamentally conserved mammalian skull morphology.

### Phenotypic impact of candidate region variation

There is a significant association between haplotype variation under a genomic region on mouse chromosome 17 and variation in the lengths of the zygomatic process of the maxilla and the zygomatic bone. While we are confident that there is a causal factor in this region, zygomatic arch element lengths are likely highly polygenic as is the norm for skull morphology [22, 38]. As with most skull bone lengths, the length of the zygomatic bone and the zygomatic process of the maxilla also correlate positively with skull size. In fact, the amount of variation explained by the correlation with skull size (as measured by *R*^2^) is greater than the amount of variation explained by the negative correlation between the two zygomatic components (Table 1). Additionally, the greatest difference between haplotype specific phenotypic effects in our genomic interval is about 0.4 mm (Fig. 4), which is approximately one standard deviation for these bone length measurements across our DO mice. Although the identified negative pleiotropy plays an important role in limiting overall zygomatic arch morphology, system-wide growth factors play a stronger role in determining all zygomatic arch bone lengths.

In addition to the interval displaying negative pleiotropy, another peak on chromosome 17 between 42 and 46 Mb met the genome-wide significance level for association with zygomatic bone length. With larger samples and different measurements of zygomatic arch morphology, other regions of interest would also be identified (as in [39]). Furthermore, although a mouse with the WSB haplotype under our interval of interest had, on average, a 0.3 mm shorter zygomatic bone than other DO mice (Fig. 4), WSB inbred founder mice don’t all have a shorter zygomatic bone than other inbred founder strains. This is because WSB alleles in other regions of the genome also contribute to the total WSB founder strain phenotype. Variation in even small-scale skull morphologies is produced by the combination of numerous factors, some acting globally across an organism and some acting more locally [11, 22]. However, determining the genetic factor on mouse chromosome 17 that drives negative pleiotropy within the zygomatic arch may help to reveal an important basis of developmental constraint and evolutionary change within the skull.

### Candidate genes

Three protein-coding genes (*Camkmt*, *Six3, and Six2*) and one non-coding RNA with known function (*Six3os1*) are found under our candidate region of interest (Fig. 6). Our real-time PCR results indirectly support *Camkmt* and *Six2* as candidate genes. Although we cannot definitively rule out other genetic factors under and near this genomic interval as candidates, we speculate that changes in the expression of at least one of these four identified factors might be responsible for the noted negative pleiotropy in zygomatic arch element length.

CAMKMT (calmodulin-lysine N-methyltransferase) is expressed across a wide range of tissues and plays a pivotal role in the methylation of calmodulin, which changes across developmental stages and varies in a tissue specific manner [40]. Deletion of a genomic region including *CAMKMT* in humans has been associated with micrognathia, dolichocephaly, and cleft palate, although the specific loss of CAMKMT has been associated with intellectual disability and muscle fiber abnormalities instead of these craniofacial phenotypes [41]. Since CAMKMT regulates calmodulin (CaM) function, it is also important to note that calmodulin has been linked to variation in beak length in Darwin’s finches and chicks [42]. CAMKMT is critical to basic physiological function across the body and has tentatively been associated with severe craniofacial birth defects.

SIX2 (sine oculus-related homeobox 2) is known to play a significant role in the skeletal development of pharyngeal arch derivatives. SIX2 is upregulated in neural crest-derived cranial mesenchyme in mice at E8.5 [43] and E9.5 [44], becoming localized to mesenchymal cells of nasal prominences, midline, and developing skull vault, as well as olfactory epithelium by E11.5 [43, 45, 46]. Downregulation of SIX2 can lead to loss or reduction in facial skeletal elements [47], reduced cranial base length, and cleft palate [48]. Later in development, SIX2 loss has been linked to increased rates of cartilage replacement by bone during endochondral ossification of the presphenoid, leading to an abnormal cranial base morphology [49]. SIX2 regulates the formation of bones from the first pharyngeal arch, which includes both the maxilla and zygomatic bones.

SIX3 (sine oculus-related homeobox 3) interacts with BMP, WNT, and NODAL, is critical during eye development [50–52], and during anterior neural plate specification [53]. Although SIX3 is not expressed in the maxillary arch or other craniofacial mesenchyme during embryonic development [44], genetic variation in *Six3* can lead to craniofacial dysmorphology of maxillofacial bones [54], particularly in the facial midline. Mutations in *SIX3* can cause holoprosencephaly in humans, a condition associated with forebrain malformation, intellectual disability, ophthalmological abnormalities, and craniofacial features including cyclopia, nasal dysmorphology, and cleft lip/palate [55]. A combination of forebrain loss and modified ectoderm/mesenchyme interactions may underlie the associated craniofacial dysmorphology (e.g., [56]). SIX3OS1 (Six3 opposite strand) is an antisense non-coding RNA that is independently coexpressed with SIX3 in the forebrain and eye after E8.5 in mice [57]. SIX3OS1 likely acts as a transcriptional scaffold for SIX3 and modulates the ability of Six3 to regulate expression of target genes in retinal cells [58]. SIX3 and SIX3OS1 may indirectly regulate facial bone development.

Although all four candidate genes have been previously associated with craniofacial dysmorphology, *Six2* is particularly tantalizing because it is a major player in facial bone ossification and is associated with RNA expression level differences between founder strain maxillary prominences. Assuming that variation in *Six2* or one of its cis-regulatory factors is responsible, we speculate about how variation in SIX2 expression might modify development to produce zygomatic arch variation in our DO mice and across mammalian clades.

### Developmental mechanisms

All bones that contribute to the mouse zygomatic arch form from neural crest-derived mesenchyme [59, 60] within the first pharyngeal arch [61–63]. The maxilla and zygomatic bone derive from neural crest cells that migrate from the posterior mesencephalic region, while much of the squamous temporal neural crest mesenchyme probably originates in the first couple of rhombomeres [5, 64]. Our results indicate that a gene or regulatory element within our candidate interval determines the location of the border between the maxilla and zygomatic within the adult zygomatic arch. Because the zygomatic bone is the last remaining dermally ossified circumorbital bone in mammals [6, 65] and appears to be the only dermatocranial element that does not develop in proximity to chondrocranial elements [66], it may ossify in response to a different signal than the maxilla. Although *Dlx* genes and related factors are associated with determining upper and lower jaw fates within the first pharyngeal arch [67], what determines the individual bone fate of mesenchymal cells within the maxillary portion of the first pharyngeal arch remains unknown.

Heuzé and colleagues [68] recently suggested that the zygomatic progenitor cells experience developmental cues analogous to *Dlx* expression patterns that distinguish them from maxillary cell populations. It is possible that one of these cues is the spatiotemporal pattern of *Six2* expression. Supporting this perspective, loss of SIX1 results in a partial transformation of the zygomatic process of the maxilla into a mandible, possibly through downstream effects on *Dlx* gene expression [69]. This partial morphological transformation includes a significant increase in the length and volume of the zygomatic process of the maxilla and a significant reduction of the zygomatic bone or the fusion of zygomatic and maxillary portions of the zygomatic arch together [69]. As two members of the same gene family that are expressed within the developing pharyngeal arches, it is possible that SIX1 and SIX2 regulate development through similar pathways and mechanisms.

**Fig. 8:**
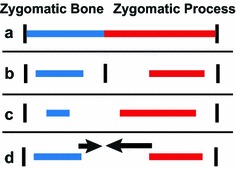
Potential developmental mechanisms underlying zygomatic arch variation. Schematic models of developmental mechanisms by which an **a** adult phenotype of relative skeletal contribution to the zygomatic arch might occur. Options include **b** external regulatory definition of a presumptive suture, **c** differences in initial mesenchymal condensation size, and **d** differences in relative growth or ossification rates between the two bone primordia

If a mutation in SIX2 leads to a change in how regional segmentation genes like the *Dlx* genes are expressed, it is possible that the variation in the contribution of maxilla and zygomatic to the zygomatic arch (Fig. 8a) may be based on a change in the location of a regulatory gene expression border between their presumptive cell populations (Fig. 8b). Tissue boundary definition is critical in many developmental contexts [70]. One well-documented craniofacial example of a gene regulatory border prevents mesenchymal and osteogenic cells from crossing the presumptive coronal suture between neural crest and mesodermally derived mesenchymal cells [70–72] (although, see [73]). A change in the relative location of a gene regulatory boundary may serve to define the final location of the zygomaticomaxillary suture.

There are other ways that a mutation in SIX2 might modify developmental processes to lead the measured zygomatic arch length variation. The site of the zygomaticomaxillary suture may not be defined prior to osteogenesis but may simply occur wherever the growing bones meet. *Six2* expression has been associated with increased mesenchymal cell proliferation in the developing head and renal system [48, 49, 74]. Recent results indicate that *Six2* mRNA and protein levels are highest in palatal tissues during the period of initial palatal shelf outgrowth and suggest that later spatiotemporal expression patterns are responsible for local increases in mesenchymal cell proliferation [48]. It is possible that genetic variation under our candidate region leads to a change in the timing, location, or level of mesenchymal precursor cell populations.

A change in proliferation within either the maxillary or zygomatic mesenchymal condensations may result in size variation of that condensation and the resulting bones (Fig. 8c). Within chicken eyes, it has been shown that the largest and most widely spaced intramembranously ossified scleral ossicles tend to be those with the earliest forming mesenchymal condensation precursors. In addition, if one ossicle fails to form, the adjacent ossicles fill in the extra space [75]. In another relevant example, a *Fuz* ciliopathy mutation leads to the formation of a single frontal bone pair at the expense of parietal bones in mice, perhaps because an excess proliferation of precursor cells leads to an unusually wide frontal bone mesenchymal condensation [76]. It is possible that a similar change in the proliferation rate within the maxillary or zygomatic bone mesenchymal condensations may cause them to become larger at the expense of the other. While *Six2* is a tantalizing candidate gene that may drive the negative pleiotropy in zygomatic arch bone lengths among DO mice, further work is required to confirm this.

### Evolutionary implications

The jugal bone (homologous with zygomatic bone) is first noted in the fossil record as one of the circumorbital bones within the dermal skeleton of agnathans. Within the presumed ancestral tetrapod, the jugal was a narrow bone of the inferior orbital margin that articulated with facial bones including the lacrimal, maxilla, and squamosal (reviewed by [65, 68]). The zygomatic bone of the last common ancestor of mammals was likely a linear bone connecting the maxilla and squamosal bones that lacked a postorbital connection to the frontal bone. This view is supported by the existence of similar morphologies in living monotremes, marsupials and many other mammalian clades. Among mammals, zygomatic arches vary in their width, breadth, height, length, degree and direction of curvature, among other characteristics. A complete postorbital border between frontal and zygomatic bones is noted in some clades (e.g., cervids, equids, primates), is sometimes represented by zygomatic and frontal processes that do not touch (e.g., carnivorans, lagomorphs), and completely absent in other mammals (e.g., bats, insectivores, rodents) [77–79]. Arch thickness and robusticity may vary in response to selective pressure based on mechanical loading requirements [80–83], with robust arches found in beaver and rabbits, thinner arches in mice and moles, and a practically complete loss in shrews.

Although a wide range of morphologies occur across mammals, our results specifically relate to variation in the relative contribution of bones to zygomatic arch length between the main bodies of the maxilla and squamosal bones. The zygomatic process of the maxilla, the zygomatic bone, and the zygomatic process of the squamous temporal typically contribute to form the zygomatic arch. Because only a short squamous temporal portion exists in laboratory mice, our analysis focused on the zygomatic and maxillary bone contributions to overall arch length. Mouse strain haplotype variation noted under a genomic interval on mouse chromosome 17 (85.3–85.9 Mb) is responsible for significant variation in the relative contribution of these bones to arch length.

**Fig. 9:**
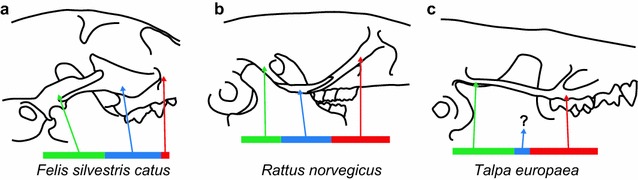
Mammal zygomatic contributions. **a** snapshot of mammalian variation in the relative contributions of zygomatic arch bones, including a feral domestic cat (*Felis silvestris catus*), **b** a wild caught common rat (*Rattus norvegicus*), and **c** a European mole (*Talpa europae*). Outlines based on images found on DigiMorph (digimorph.org). Colored lines representing the relative length of the zygomatic process of the maxilla (red), the zygomatic bone (blue), and the zygomatic process of the squamous temporal (green) match the colors noted in Figs. 2 and 8

Analogous genetic variation may underlie variation in the relative contribution of zygomatic arch elements to total arch length across mammalian species. This sort of phenotypic variation is common across mammalian taxa. For example, carnivorans including felids and canids typically have a very short zygomatic process of the maxilla, an anteriorly placed zygomatic bone, and a relatively long zygomatic process of the temporal [78, 84] (Fig. 9a). On the other hand, rodents typically have a long zygomatic process of the maxilla, a more posteriorly placed zygomatic bone, and a short zygomatic process of the temporal [78, 85] (Fig. 9b). We propose that the identified pattern of negative pleiotropy contributes to this mammalian variation in relative bone length, but not that it explains all variation in zygomatic arch length or shape.

Two clades that were commonly thought to lack a zygomatic bone illustrate how an extreme imbalance in two zygomatic arch elements might manifest. First, moles (Talpidae) may represent the logical extreme of the proposed mechanism. Although moles lack a separate zygomatic bone as adults [78, 86] (Fig. 9c), one of the multiple small zygomatic arch ossification centers [77] may represent the zygomatic bone [87] and fuse with the zygomatic process of the maxilla quite early in development. The mole zygomatic arch is a complete arch, with a minor zygomatic bone contribution, and a lack of zygomatic arch sutures. The fossil order of Multiturberculata was long considered unique as mammals with a robust zygomatic arch, but lacking a zygomatic bone. However, careful work by Hopson and colleagues [88] indicated the zygomatic bone is found medial to the maxillary and temporal portions of the arch rather than in between.

If the modified expression of a factor like SIX2 leads to the expansion of one arch bone at the expense of another, a fusion of the arch bones together and/or a displacement of the smaller bone may occur. In fact, SIX1 null mutant mice with the enlarged zygomatic process of the maxilla also display either a smaller displaced zygomatic bone or fusion of zygomatic and maxillary portions of the developing arch [69]. It is temping to speculate that this reduction and fusion of the zygomatic bone might serve as a foundation for total zygomatic bone loss. In moles, where the existence of a zygomaticomaxillary suture is not functionally necessary, a complete loss of the zygomatic bone ossification center would result in the same morphology as long as the growing temporal and maxillary bones expanded further to fill in the gap as occurs among chick scleral ossicles [75]. However, in the case of the Multiturberculata, the continued presence of the displaced zygomatic bone may serve to reinforce the zygomatic arch response to mechanical forces [88]. Regardless of the responsible factor, similar changes in developmental processes [89] may underlie the variation in zygomatic arch bone contributions among our DO mice and variation that has arisen during mammalian evolution.

## Conclusions

Investigating how genetic factors constrain the range of possible skull variation is critical for identifying mechanisms of integration and developmental constraint. Out of three cranial regions studied, we identified a genomic region underlying negative pleiotropy within the zygomatic arch. Association mapping and subsequent RT-PCR analysis identified candidate genes that might underlie this pattern. Further study is required to determine how the responsible genetic factor modifies developmental processes to limit overall zygomatic arch length while allowing for variation in the relative length of contributing zygomatic bones. This pattern of negative pleiotropy may have contributed to the evolution of mammalian zygomatic arch diversity. Identifying the particular developmental basis for this negative pleiotropy has implications beyond the zygomatic arch, because changes in similar instances of negative pleiotropy underlie significant evolutionary variation in the relative contribution of adjacent bones to larger morphological features in other regions like the cranial vault and upper jaw. These results provide a significant toe-hold in unraveling an example of negative pleiotropy and developmental constraint, which is an important step toward understanding the developmental basis for evolutionary change in the skull.

## Additional files

**Additional file 1.** A.csv file with sequence details and identification of primers used during RT-PCR analysis.

**Additional file 2.** *Validation Sample Haplotype Effects* CC founder strain specific phenotype coefficients (above) and LOD scores from genome-wide scan (below) for significant association between haplotype and A) zygomatic process of the maxilla length and B) zygomatic bone length across Chromosome 17. Phenotypic coefficients are the effect of having a certain founder strain genotype at a specific genomic location on a measurement.

**Additional file 3.** A.csv phenotype file with linear distance measures and covariates for the CC Parental/F1 sample discussed in this analysis.

**Additional file 4.** A.csv phenotype file with linear distance measures and covariates for the primary DO sample discussed in this analysis.

**Additional file 5.** A.csv phenotype file with linear distance measures and covariates for the validation DO sample discussed in this analysis.
