# Supplementary figures and images for: Developmental constraint through negative pleiotropy in the zygomatic arch
Source: EvoDevo. 2018 Jan 27;9:3. doi: 10.1186/s13227-018-0092-3 (PMC5787316; doi:10.1186/s13227-018-0092-3)

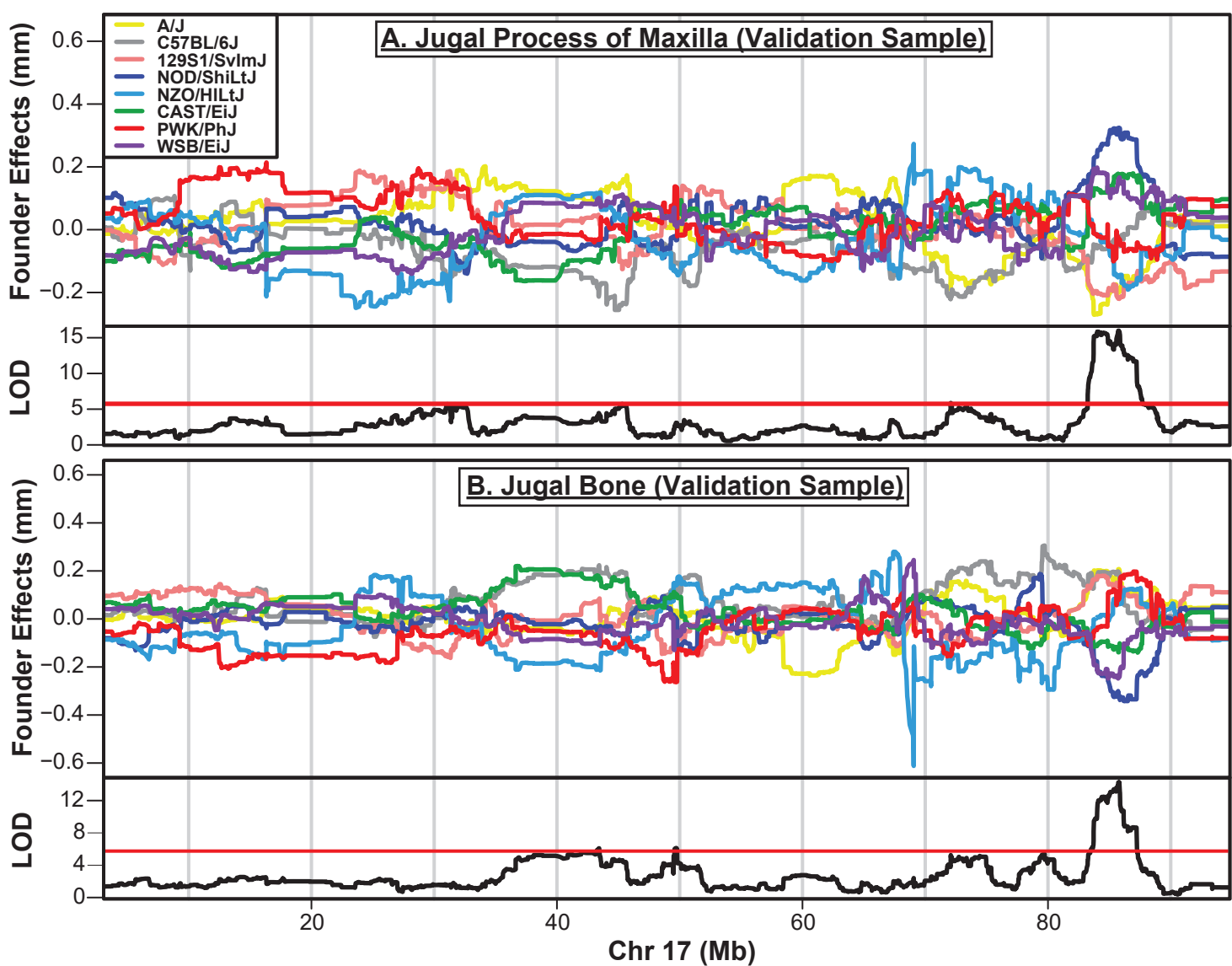

Supplement: Supplementary file 2 — Additional file 2. Validation Sample Haplotype Effects CC founder strain specific phenotype coefficients (above) and LOD scores from genome-wide scan (below) for significant association between haplotype and A) zygomatic process of the maxilla length and B) zygomatic bone length across Chromosome 17. Phenotypic coefficients are the effect of having a certain founder strain genotype at a specific genomic location on a measurement. [file 13227_2018_92_MOESM2_ESM.pdf]
